# A Mini-Review of Strategies for Quantifying Anthropogenic Activities in Microplastic Studies in Aquatic Environments

**DOI:** 10.3390/polym14010198

**Published:** 2022-01-04

**Authors:** Chun-Ting Lin, Ming-Chih Chiu, Mei-Hwa Kuo

**Affiliations:** Department of Entomology, National Chung Hsing University, Taichung 40227, Taiwan; b03612016@g.ntu.edu.tw

**Keywords:** anthropogenic activities, microplastics, quantification, freshwater, marine

## Abstract

Microplastic pollution is no longer neglected worldwide, as recent studies have unveiled its potential harm to ecosystems and, even worse, to human health. Numerous studies have documented the ubiquity of microplastics, reflecting the necessity of formulating corresponding policies to mitigate the accumulation of microplastics in natural environments. Although anthropogenic activities are generally acknowledged as the primary source of microplastics, a robust approach to identify sources of microplastics is needed to provide scientific suggestions for practical policymaking. This review elucidates recent microplastic studies on various approaches for quantifying or reflecting the degree to which anthropogenic activities contribute to microplastic pollution. Population density (i.e., often used to quantify anthropogenic activities) was not always significantly correlated with microplastic abundance. Furthermore, this review argues that considering potential sources near sample sites as characteristics that may serve to predict the spatial distribution of microplastics in aquatic environments is equivocal. In this vein, a watershed-scale measure that uses land-cover datasets to calculate different percentages of land use in the watershed margins delineated by using Geographic Information System (GIS) software is discussed and suggested. Progress in strategies for quantifying anthropogenic activities is important for guiding future microplastic research and developing effective management policies to prevent microplastic contamination in aquatic ecosystems.

## 1. Introduction

The term “microplastic”, which refers to tiny debris of plastics normally defined to be smaller than 5 mm [1], was not widely used until 2004 [2]. Approximately 10% of municipal waste globally comprises plastics [3]. The vast use of plastic in human life has resulted in the ubiquity of microplastics in the environment, as they can be degraded into small, persistent, and therefore easy-to-transport plastic debris [4]. For example, microplastics have been detected in a variety of environments, such as beaches, bays, estuaries [5], ocean surfaces [6], deep-sea sediments [7], rivers [8], lakes [9], raindrop [10], the Alps and the Arctic [11], and polar waters [12]. Microplastics have also been documented in biota, including riverine macroinvertebrates [13], marine fish [14], and birds [15]. The accumulation of microplastic pollution is considered an environmental hazard that has attracted global concern. Generally, microplastics originating from terrestrial environments are either retained in freshwater systems or eventually enter the ocean [16]. In this context, this review mainly focuses on aquatic environments.

Many studies have discussed the impact of microplastics on organisms [17], and these impacts can be categorized into two types: physical and chemical [18]. Physical impacts can then be categorized as being either direct or indirect. The direct impacts of microplastics have been observed in numerous studies [17], as ecotoxicological assessments of microplastic pollution are frequently conducted on different species in the laboratory. Generally, the detrimental consequence of microplastic ingestion results from the blockage of the digestive system, which reduces nutrition intake, inhibits food assimilation [19], and causes inflammation [20], resulting in the reduction of growth, reproduction, fitness, mortality, emergence delay, and immune-system weakening [18,21]. Furthermore, indirect impacts of microplastic pollution on organisms also occur. These detrimental effects are not caused by the ingestion of microplastics per se but include the alteration of gut microbiota [22], induction of microbiota dysbiosis [23], ecosystem functioning change [24], behavioral change [25], and locomotion interruption [26]. Plastisphere, a term denoting the microbiome of microplastics, has raised global concern because its community structures are distinct from the natural environment. *Vibrio*, a genus of bacteria, is represented in the plastisphere of the North and Baltic Sea [27], and can have harmful effects on the human body [28].

Chemical impacts are caused by chemical additive consumptions, which are added to plastics during their production, and organic pollutants, which tend to attach to microplastics because of their large surface area to volume ratio [29]. These chemical substances can be easily exposed [30], especially under ultraviolet radiation and extreme heat [31,32]. For example, plasticizers added to plastic products for flexibility and malleability enhancement are not stable and can leach into the environment [33]. Additives such as bisphenol A (BPA), polybrominated diphenyl ethers (PBDEs), and phthalates are also known as endocrine-disrupting compounds (EDCs) and are harmful to the endocrine system [31]; these directly (reception of plasticizers by hormone receptors on microbes [34]), and indirectly (interruption of host hormone signaling) influence gut microbes, as gut microbes are mediated by hormones secreted by their hosts [29].

In summary, microplastics or substances attached to them can induce immediate and chronic mechanical and chemical disruptions in organisms. Preventing microplastic pollution of natural habitats is necessary to overcome these problems. Therefore, identifying microplastic sources is imperative to mitigating this damage. However, most review articles mainly focus on the risks of microplastics to organisms; the methodological progress of microplastic extraction and identification; and the comparison of microplastic occurrence, size, shape, type, color, and abundance between publications [35,36,37]. Discussions on how these reports attributed microplastic pollution to various anthropogenic factors have been limited. This review aims to elucidate the current advancements in the strategies used to analyze the relationship between anthropogenic activities and microplastic pollution.

## 2. Microplastics and Anthropogenic Activities

**Figure 1:**
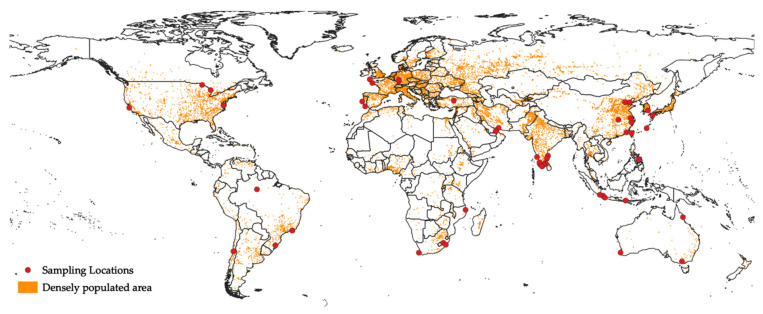
Distribution of the sampling sites of studies that linked microplastic pollution to anthropogenic activities. Densely populated area data were retrieved from Natural Earth (http://www.naturalearthdata.com (accessed on 20 December 2021) [43]. The Antarctica region was excluded as it is an area with limited human activities. Coordinate reference system: WGS 84, EPSG: 4326.

The major sources of microplastics are anthropogenic activities, such as human manufacturing and plastic-product usage. Humans are a major source of microplastics. The increasing world population size is a possible reason for the increasing plastic waste [2,38,39], owing to the short lifetime that these plastics are actually in use [40]. In 2019, while the world population reached 7700 million [41], the enormous demand for plastic drove the world plastic production up to 370 million metric tons a year [42], which has attracted attention as the growing rate of plastic recycling is overtaken by the growing rate of plastic production. Although the recycling rate of plastic waste from 2006 to 2018 has doubled, 25% of plastic waste is still sent to landfills [42]. Furthermore, since the COVID-19 pandemic happened, the relationship between anthropogenic activities and microplastics has become clearer. The plastic demand decreased tremendously during the pandemic in Europe in 2020, due to the quarantine, indicating less human activity, and therefore less plastic production [42]. However, the subsequent lifting of the lockdown restriction implies a resumption of plastic demand, and thus the microplastic problem remains to be solved. In this section, we introduce global publications (*n* = 34) that have linked microplastic abundance to potential anthropogenic factors (Figure 1), with Europe, India, and China being the top three most studied regions. Indeed, studies on the relationship between human activities and microplastic pollution in many densely populated areas are still in the developing stage, and providing in-depth focus on the link between these variables is necessary for studies that examine microplastic pollution as a function of spatial factors. Therefore, this review aims to not only amplify the importance of defining the relationships between variables, but also to explain why a better measure than population density for quantifying anthropogenic activities is needed and why statistical analysis is essential.

### 2.1. Population Density

Numerous studies have shown that areas with intensive anthropogenic activities tend to have higher microplastic pollution levels [17,44,45,46,47,48,49,50]. Previous reports related to aquatic environments (*n* = 34) are listed in Table 1, showing that 64.7% of studies sampled microplastics from water surface/column, 61.8% sampled microplastics from sediments, and only 29.4% sampled microplastics from organisms. Above all, only 50% of studies have conducted statistical analyses to investigate the relationship between anthropogenic factors and microplastic abundance, while 45, 50 and 50% of studies made statistical conclusions regarding the relationship between the two in water surfaces/columns, sediments, and organisms, respectively. Such paucity underlines the pressing need to conduct more statistics-based research in this field, and only half of the studies addressing the relationship between microplastic and anthropogenic activities is insufficient to formulate reliable microplastic control policies. Indeed, because of the heterogeneity of anthropogenic activities, previous studies have usually treated anthropogenic activities as a point source of microplastics. Those studies reflected the degree to which anthropogenic activities were responsible, mainly with regards to population density and proximity to city centers, wastewater treatment plants (WWTPs), harbors, and highly urbanized areas [5,9,44,45,51].

Browne et al. [45], for example, investigated microplastic pollution in sediments sampled from 18 sandy beaches worldwide, with microplastic abundance ranging from 2 to 31 particles in 250 mL sediment, suggesting that population density is significantly positively correlated with level of microplastic pollution (*p* < 0.05, r^2^ = 0.34). However, it was difficult to compare this study with other sediment-focused microplastic studies on the coastline, as most relevant studies used weight/area rather than volume as the sampling unit. Yonkos et al. [52] supported this conclusion, demonstrating that variation in microplastic abundance on sampling dates (5534 to 297,927 particles km^−^^2^) at the water surface of a bay was significantly correlated with population density (*p* < 0.05, r^2^ = 0.33). In addition, Tang et al. [53] also suggested that, when their observations (514 particles m^−^^3^ on average) were integrated with other studies that took place in coastal areas of China, microplastic abundance at the water surface was significantly correlated with population size (*p* < 0.05, r^2^ = 0.99) and urbanization rate (*p* < 0.05, r^2^ = 0.98). Compared with not only a bay in South Korea, where the abundance at the water surface was 770 particles m^−^^3^ on average [47], but also other reports in China (see Reference [53]), the abundance observed by Tang et al. [53] was lower. This was possibly due to (1) different sampling methodologies, (2) different degrees of population density in sampling sites, and (3) samples being collected during the rainy season. More importantly, microplastic abundance in urban areas was not significantly different from that in rural areas with low population density (ANOVA, *p* > 0.05) in a bay in South Korea [47]. Furthermore, Wang et al. [51] found that, in China, distance from Wuhan City Center was significantly negatively correlated with microplastic abundance (*p* < 0.05, r^2^ = 0.90), indicating a close relationship between human activities and microplastic pollution. Similarly, microplastic abundance in sediment (11 to 234.6 particles kg^−1^) in heavily polluted areas in Taihu Lake, based on the index of eutrophication that generally reflects the degree of anthropogenic activities, was significantly higher than it was in clean areas (ANOVA, *p* < 0.05) [9].

In contrast, many studies provided no evidence of a relationship between population density and microplastics, as population density was not significantly associated with microplastic concentration [38,50,54]. For example, no significant relationship was found between the local municipal population and the level of microplastic abundance in water (*p* > 0.05) and sediment (*p* > 0.05) in the South African coastline, although some harbors had significantly higher microplastic loads (up to 1200 particles m^−^^3^) in the water column (ANOVA, *p* < 0.05) [38]. Furthermore, Townsend et al. [50] investigated microplastic abundance in the wetlands in Australia (2 to 147 particles kg^−^^1^), suggesting that neither population size (*p* > 0.05) nor population density (*p* > 0.05) was significantly correlated with microplastic abundance. Klein et al. [55] analyzed microplastics in river-shore sediments in Germany (228 to 3763 particles kg^−1^), suggesting that population density was not significantly correlated with microplastic abundance (*p* > 0.05), and similarly microplastic abundance did not vary as a function of proximity to industrial areas or wastewater treatment plants. This disparity indicates that neither population density, a measure to quantify anthropogenic activities as a point source of microplastics, nor the characteristics of sample sites and their surroundings can fully explain the spatial variability of microplastics, with the latter measure being common in previous reports (see next section).

**Table 1:** Sampling condition, quantitative data and quantification strategies of anthropogenic activities in microplastic (MP) studies in aquatic environments. Note: dw, dry weight; ww, wet weight.

Environment	Sample Type and Average MP Concentration for Sampling Sites				Statistical Analysis	Anthropogenic Factors	Conclusion	Reference
	Water Surface	Water Column	Sediment	Organism				
Bay	0.24 ± 0.35 MP m^−3^ (excluding fibers, mean ± SD)	-	0.97 ± 2.08 MP kg^−1^ (excluding fibers, dw, mean ± SD)	-	-	1. Commercial port 2. Military base 3. Wastewater treatment plant 4. Shellfish farming 5. Marina	MP abundance at water surface was higher in sites next to anthropogenic factors	[5]
Bay	2.2 ± 1.4 MP L^−1^	1.6 to 6.9 MP L^−1^	31.1 to 256.3 MP kg^−1^ (dw)	-	-	1. Vessel activity 2. Close to coastline	MP abundance at water surface and in columns was higher in sites next to anthropogenic factors	[56]
Bay	7.62 MP m^−3^	-	Beach: 166.50 MP kg^−1^ bay sediment: 20.74 166.5 MP kg^−1^	-	-	1. Aquaculture 2. Fishing activity 3. recreational activities 4. Marine sports activities 5. Bars and restaurants 6. Proximity to rivers and channels 7. Urban drainage 8. Boat marina 9. Proximity to roads and waterway transport	MP abundance might be related to adjacent potential human activities, greater river inflow, and lower hydrodynamics	[57]
Bay	For each sampling date: 5534 to 297,927 MP km^−2^ or 2.7 to 245.7 g km^−2^	-	-	-	V	1. Land use (proportion of urban/suburban area, agricultural area in catchments) 2. Population density	MP abundance was significantly correlated with population density and the proportion of urban/suburban development in the catchment	[52]
Bay	-	-	In Lumpung: 72.64 ± 25.28 MP kg^−1^ (mean ± SD) In Sumbawa: 44.19 ± 12.40 MP kg^−1^ (mean ± SD)	Sandfish: In Lampung: 3.21 ± 1.07 MP fish^−1^ (mean ± SD) or 126.34 ± 51.99 MP kg^−1^ (mean ± SD) In Sumbawa: 1.39 ± 0.86 MP fish^−1^ (mean ± SD) or 69.69 ± 52.22 MP kg^−1^ (mean ± SD)	V	1. Populated area	MP abundance in sediment and sandfish was significantly higher in Lumpung (populated area) than in Sumbawa (semi-enclosed ecosystem).	[58]
Bay/Coastline	0.77 ± 0.88 MP L^−1^ (mean ± SD)	-	0.94 ± 0.69 MP g^−1^ (ww, mean ± SD)	Mussel: 1.43 ± 1.45 MP g^−1^ (ww, mean ± SD) Oyster: 1.13 ± 0.84 MP g^−1^ (ww, mean ± SD) Polychaete: 0.71 ± 1.00 MP g^−1^ (ww, mean ± SD)	V	1. Close to urban areas 2. Close to aquafarm areas	MP abundance in sediment was significantly higher in urban areas than in rural areas	[47]
Bay/Coastline/Estuary	514.3 ± 520.0 MP m^−3^ (mean ± SD)	-	-	76 to 333 MP kg^−1^(mean ± SD)	V	1. Total population 2. Urbanization rate 3. Farmland	MP abundance at water surface was significantly correlated with total population and urbanization rate	[53]
River	-	-	Summer: 6.3 ± 4.3 MP kg^−1^ (dw, mean ± SD) Winter: 160.1 ± 139.5 MP kg^−1^ (dw, mean ± SD)	*Chironomus* spp.: Summer: 0.37 ± 0.44 MP mg^−1^ (ww, mean ± SD) Winter: 1.12 ± 1.19 MP mg^−1^ (ww, mean ± SD)	-	1. Close to populated areas 2. Close to wastewater treatment plants	MP abundance was higher in sites next to anthropogenic factors	[59]
River	892,777 MP km^−2^	-	-	-	-	1. Close to populated areas 2. Close to wastewater treatment plants	MP abundance was higher near populated areas and at the side of riverbanks wherein wastewater treatment plant effluents are entering	[60]
River	-	-	0.063–5 mm: 417 to 8178 MP kg^−1^ (dw) 0.063–1 mm: 0 to 5725 MP kg^−1^ (dw)	-	-	1. City	Location with highest MP concentration might be related to hydraulic conditions and proximity to the city	[61]
River	Reference area: 6.8 MP L^−1^ Textile industrial area: 13.3 MP L^−1^	-	16.7 to 1323.3 MP kg^−1^ (dw)	-	V	1. Close to textile industrial area	MP abundance was significantly higher in the industrial area than in the reference area	[46]
River	9.2 ± 2.2 MP L^−1^ (mean ± SD)	Intermediate: 8.4 ± 1.7 MP L^−1^ (mean ± SD) Bottom: 14.2 ± 5.6 MP L^−1^ (mean ± SD)	4328 ± 2037 MP kg^−1^ (dw, mean ± SD)	-	V	1. Population density of suburban area 2. Population density of urban area 3. Population density of industrial area	MP abundance in water columns was significantly correlated with population density in suburban and urban areas	[48]
River	-	-	Rhine river: 21.8 to 932 mg kg^−1^ or 228 to 3763 MP kg^−1^ Main river: 43.5 to 459 mg kg^−1^ or 786 to 1368 MP kg^−1^	-	V	1. Close to industrial area 2. Population density	No significant correlation between MP masses and population density was found, and MP abundance did not increase downstream of the industrial area	[55]
River	-	-	-	Chironomidae larvae: 0.28 to 2.07 MP mg^−1^	V	1. Land use (proportion of industrial area and residential area in the catchment)	The proportion of industrial areas in catchment contributes more to MP concentration in midge larvae than the proportion of residential areas	[49]
River	-	5.85 ± 3.28 MP L^−1^ (mean ± SD)	3.03 ± 1.59 MP 100 g^−1^ (dw, mean ± SD)	-	V	1. Industrial area 2. Slum area	MP abundance in sediment was significantly higher in sites located around industrial and slum areas	[62]
River/ Coastline	8.48 to 9.37 MP m^−3^	-	-	*Aplocheilus* sp.: 1.97 MP fish^−1^	-	1. Tourism 2. Port 3. Industrial operation	MP abundance was higher in sites located around anthropogenic factors	[63]
River/Lake	-	1660.0 to 8925 MP m^−3^	-	-	V	1. Distance from the urban left	MP abundance correlated significantly negatively with distance from the city left	[51]
Lake	43,157 ± 115,519 MP km^−2^ (mean ± SD)	-	-	-	-	1. Close to populated areas 2. Close to shoreline	MP abundance was higher near populated areas and areas near the shoreline	[64]
Lake	11.9 to 61.2 MP m^−3^	-	-	-	-	1. Population density 2. Domestic sewage	MP abundance was higher in sites located around populated area	[65]
Lake	3.4 to 25.8 MP L^−1^	-	11.0 to 234.6 MP kg^−1^ (dw)	Plankton: 0.01 × 10^6^ to 6.8 × 10^6^ MP km^−2^ Asian clams: August: 1.3 to 12.5 MP g^−1^ (ww) November: 0.2 to 9.6 MP g^−1^ (ww)	V	1. Close to populated areas 2. Index of eutrophication	MP abundance in sediment was significantly higher near areas with more human activity than areas with less human activity, according to the index of eutrophication	[9]
Lake	0.05 to 32 MP m^−3^	-	-	-	V	1. Land use (proportion of industrial area, agricultural area (total, crops, pasture, and hay) and impervious area) 2. Population density 3. Wastewater treatment plant effluent contribution	MP abundance was significantly correlated with the proportion of urban area, agricultural area (total and crops), and impervious area in catchments; MP abundance was significantly correlated with population density	[66]
Coastline	-	-	High tide line: 439 ± 172 to 119 ± 72 MP kg^−1^ (dw, mean ± SD) Low tide line: 179 ± 68 to 33 ± 30 MP kg^−1^ (dw, mean ± SD)	-	-	1. Metropolitan city	MP abundance was highest in the location near the metropolitan city	[67]
Coastline	-	24 ± 9 to 96 ± 57 MP L^−1^ (mean ± SD)	55 ± 21 to 259 ± 88 MP kg^−1^ (mean ± SD)	-	-	1. Tourism 2. Shipping 3. Fishing 4. Aquaculture	MP abundance was higher in sites located around anthropogenic factors	[68]
Coastline	-	3.1 ± 2.3 to 23.7 ± 4.2 MP L^−1^ (mean ± SD)	-	0.11 ± 0.06 to 3.64 ± 1.7 MP fish^−1^ (mean ± SD) or 0.0002 ± 0.0001 to 0.2 ± 0.03 MP g^−1^_gut weight_ (mean ± SD)	-	1. Sewage effluent 2. Proximity to anthropogenic activities	MP abundance was higher in shore areas (adjacent to sewage effluent) and in epipelagic fish (adjacent to urban runoff)	[69]
Coastline	-	1.25 ± 0.88 MP m^−3^ (mean ± SD)	40.7 ± 33.2 MP m^−2^ (mean ± SD)	Fishes (not specified)	-	1. Population density 2. Industrial activities 3. Tourism 4. Sewage effluent 5. fishing	MP abundance in water and sediment was high due to proximity of urban regions, river runoff, fisheries and tourism	[70]
Coastline	-	-	-	Zooplankton: 0.002 to 0.036 MP m^−3^	-	1. Close to populated areas 2. Close to industrial facilities 3. Close to port facilities	MP abundance was higher near populated areas and areas close to industrial and port facilities	[71]
Coastline	-	-	43 MP 50 g^−1^ (dw, only include fragments and fibers)	-	-	1. Tourism 2. Harbor 3. Residential area	MP abundance was high in beaches with associated anthropogenic activity	[72]
Coastline	-	-	2 to 31 MP 250 mL^−1^	-	V	1. Population density	MP abundance was significantly correlated with population density	[45]
Coastline	-	(Not specified)	86.67 ± 48.68 to 754.7 ± 393 MP m^−2^ (depth 5 cm, mean ± SD)	-	V	1. Close to harbors 2. Population density	No significant correlation between population density and MP abundance in water column and sediment was found	[38]
Coastline	-	-	High tide line: 1323 ± 1228 mg m−^2^ (mean ± SD) Low tide line: 180 ± 261 mg m−^2^ (mean ± SD) Overall: 46.6 ± 37.2MP m^−2^ (mean ± SD)	Important fish species: 0.1 MP fish^−1^	V	1. Tourism 2. Fishing 3. River mouth 4. Urban activities	MP abundance in beaches was insignificantly correlated with the distance of the beach from the nearest river mouth	[73]
Coastline	Proportion of MP in collected particles: 13.3 to 25.0%	-	-	-	V	1. Population density	Significantly greater proportions of MP particles were found in areas with higher population density	[74]
Pond	233 MP m^−3^	-	-	-	-	1. Populated area	MP abundance was low in the studied area (near protected areas) compared to reference study sites (near populated areas)	[75]
Strait	-	-	2 to 1258 MP kg^−1^ (dw)	-	-	1. The relative level of industrialization (manufacturing, oil refineries, and industrial sewage) and urbanization	MP abundance was higher near areas with elevated levels of industrialization and urbanization	[44]
Wetland	-	-	2 to 147 MP kg^−1^ (dw)	-	V	1. Land use (proportion of and absolute commercial area, industrial area, and residential area) 2. Dwelling density 3. Population density 4. Population size 5. Road/rail 6. Urban growth	MP abundance was significantly less in catchments with more open space (undeveloped catchments) The proportion of road/rail areas, commercial areas, industrial areas, and residential areas in catchments was not significantly associated with MP abundance Population density, population size, dwelling density, urban growth, and catchment size were not significantly associated with MP abundance	[50]

### 2.2. Importance of Statistical Analysis

It is very common to relate the effects of human activities to microplastic abundance without clear statistical analyses [5,55,56]. Previous studies tended to attribute the elevated microplastic abundance to the surrounding possible point source of microplastics, probably because it is straightforward and intuitive to infer the relationship between anthropogenic factors and microplastic abundance by associating the spatial distribution of microplastic abundance with general characterization around sample locations.

For example, although Klein et al. [55] suggested that, as mentioned above, it was difficult to visualize the relationship between microplastic abundance and proximity to industrial areas or wastewater treatment plants on a map; sample sites that were close to nature reserves had low microplastic abundance, which probably could be explained by the fewer human activities in nature reserves. In contrast, areas exhibiting high microplastic abundance on the water surface probably resulted from the proximity to marinas, military, and commercial harbors, as well as effluent from wastewater treatment plants that process sewage from more than 134,377 people [5]. In addition, we must acknowledge that those areas are located in the most densely urbanized area in the monitored region (Bay of Brest, France) [5].

Furthermore, sample sites located on the cruise route had higher microplastic abundance, supporting the inference that vessel activities produce microplastic pollution [56,76]. In addition, since certain sample sites located downstream of wastewater treatment plants showed high microplastic abundance, especially at the right river bank, and that the outlets of wastewater treatment plants entered the Rhine River from the right river bank, it can be inferred that the elevated microplastic concentration on the river surface probably resulted from the outlets of the wastewater treatment plant [60]. Additionally, consistently high microplastic abundance on the surface of Lake Erie of the Laurentian Great Lakes might be due to anthropogenic activities, as Lake Erie was the most populated lake in the monitored region [64].

In summary, reports regarding anthropogenic activities and microplastics in the field can generally be presented in two ways, depending on whether the discussion is based on statistical analyses. If yes, there were usually two kinds of mathematical results: microplastic abundance in densely urbanized areas was significantly different from that in less developed areas (reference area) [9,46,47], and there was a correlation between population density and microplastic abundance in sample sites [52,53,55]. If not, the discussion was usually made by visual inspection of anthropogenic factors surrounding the sample sites, and this can be problematic.

Microplastic distribution and abundance in monitored regions do not always depend on surrounding anthropogenic activities (e.g., location of WWTPs and harbors). According to Klein et al. [55], the four sample sites with the highest microplastic abundance, regardless of count (particles kg^−^^1^) or mass (mg kg^−^^1^), were also categorized as the four most populated sites in the research area; therefore, there is a trend indicating that population density can explain the high level of microplastic pollution at these sites. However, statistical analysis revealed no significant correlation between microplastic abundance and population density when all sample sites were considered. This highlights the potential scale-dependent effect on the results and the necessity of conducting appropriate statistical analyses to account for this. Linking these variables based on visual inspection of spatial distribution may lead to problematic conclusions. Therefore, in order to apply statistical analysis and produce practical results, two questions remain to be answered: (1) How can anthropogenic factors be quantified? (2) Are there other quantification strategies more appropriate than population density?

### 2.3. Urban Attributes

Quantifying the level of human activity by population density is simple. As mentioned above, previous studies revealed that the correlation between human activities and the level of microplastic pollution is mostly significant; on the other hand, remote and/or less developed areas showed significantly lower microplastic abundance than urbanized areas. These results, however, oversimplified anthropogenic activities and thus cannot help governments construct effective policies for controlling microplastic pollution. In other words, the anthropogenic activities that contribute microplastics to the environment predominantly remain unknown, leading to a difficult situation in which controlling microplastics from the source is the most effective way to reduce microplastics [77]. While it is no secret that human activities are the biggest source of microplastics, we still have no clue what the exact source is. There is an urgent need for detailed information on human activities.

**Figure 2:**
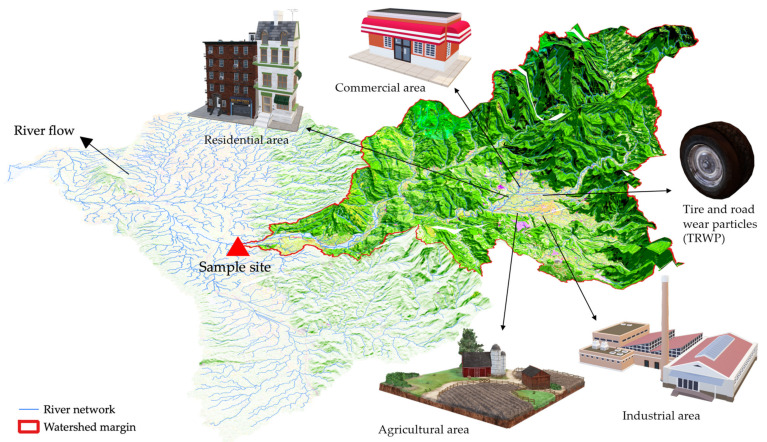
Watershed margin delineation of sample sites and different upstream land covers. Three-dimensional objects were retrieved from Microsoft^®^ Office PowerPoint^®^.

Therefore, in addition to population density, recent studies have used other urban attributes to quantify different human activities, i.e., different land uses within the catchment of the sample location (Figure 2). Figure 2 visualizes the quantification strategy of different anthropogenic factors: delineation of the catchment margins of sample sites and calculation of the percentages of different upstream land covers (e.g., industrial area, residential area, and agricultural area) in the watershed. These percentages of land cover were used to reflect the magnitude of different anthropogenic activities. For example, Yonkos et al. [52] extrapolated not only the population density in catchments of sample locations from the 2010 US census data, but also the percentages of urban (industrial), suburban (residential), agricultural, and forested areas in catchments of sample sites from the 2006 National Land Cover Database. The study estimated the correlation between different land covers and microplastic abundance and concluded that the microplastic abundance on the water surface was significantly associated with population density, percentage of urban (industrial) area, and percentage of total developed (industrial and residential) areas.

Correspondingly, Baldwin et al. [66] analyzed the correlation between the microplastic abundance on the lake surface and different watershed characteristics, including percentages of impervious areas (e.g., roads, parking lots, and buildings), urban area, agricultural areas (total, crops, pasture, and hay), and forested area in catchments of sample sites. Land-cover datasets were retrieved from the National Land Cover Database (NLCD) Land Cover and Percent Developed Imperviousness datasets, and watershed margins were derived from the US Geological Survey Watershed Boundary Dataset (USGC-WBD). The results suggested that microplastic abundance was positively correlated with the percentage of urban area and percentage of impervious areas, and negatively correlated with the percentage of agricultural area (total and crops) [66], in line with the results of Yonkos et al. [52]. Although agricultural activities were not addressed too much in the study by Baldwin et al. [66], Yonkos et al. [52] found a similar but insignificant trend in the percentage of agricultural area, which was also dominated by crop agriculture; it was negatively associated with microplastic abundance. This can probably be explained by the lower development and lesser amount of human activities in areas with high agricultural activity.

A similar approach for the quantification of human activities was conducted in the urban wetlands of Melbourne, Australia. Briefly, the study calculated the catchment margins of sample sites with certain digital elevation models, using ArcGIS 10.3 [50]. Detailed land-use data were retrieved from the 2011 Australian Population Census, including the percentages of the following: commercial area, industrial area, undeveloped area, road/rail, residential area, percentage of rural area, and semi-rural area. Additionally, urban growth and dwelling density were also included in the analyses to reflect the different magnitudes of anthropogenic activities.

However, in contrast with the study by Yonkos et al. [52], Townsend et al. [50] indicated that only the percentage of undeveloped areas within the catchment was significantly negatively correlated with microplastic abundance in sediment. No significant correlation between the percentage of industrial area and microplastic abundance in the catchment was observed. More interestingly, if not using the percentage of land use, no significant correlation was found between microplastic abundance and the absolute area of different land use, presumably resulting from the effect of catchment size. Lin et al. [49] supported this result, as the percentage of the industrial area model was better than its logarithmic model. To elaborate further, Lin et al. [49] constructed several general linear mixed-effect models and conducted model selection to investigate the effects of different land uses (industrial and residential) on microplastic concentration found in chironomid larvae. The results showed that the percentage of industrial area in catchments contributes more to microplastic concentration, a finding that is in line with previous studies that showed that the percentage of industrial (urban) areas in catchments was a potential predictor of microplastic pollution [52,66].

## 3. Future Directions and Conclusions

To make progress in mitigating environmental microplastics, the source of microplastics needs to be identified. While anthropogenic activities are the most well-known source of microplastics, one should keep in mind that quantifying parameters, such as population density, might not be detailed enough to offer practical suggestions on formulating policies for microplastic pollution management. In fact, due to the environmental risks of microplastics, government agencies and environmental protection organizations have actively advocated policies and regulations to protect aquatic organisms from the detrimental effects of microplastics [78,79]. Regulation that bans plastic/microplastic production and consumption has been articulated globally in the past few years [80]. For instance, since 2003, the government of South Africa has charged for the use of thick plastic bags, and plastic-bag use has been decreased by 90%. Since 2007, Kenya has banned the manufacture and import of thin plastic bags; however, the ban was not enforced. Since 2008, Rwanda has become the first plastic-free country by banning non-biodegradable plastics. In the same year in China, while the Beijing Olympics were in full swing, plastic bags under a certain thickness were banned, and citizens were charged for the use of others, leading to effective results in mitigating damage caused by plastic bags. In 2013, Pucón was the first city in Chili to ban plastic bags. In 2014, California, USA, banned plastic shopping bags and plastic bottles, and France implemented a tax on non-biodegradable plastic bags. In 2015, an amendment bill in the United States against personal-care products containing microbeads was passed. Similar acts have been adopted in Canada [81] and Taiwan [49]. In addition, plastic regulation in India, the most populated country and the largest plastic consumers in the world, was not successful, due to poor enforcement and pressure from the rapidly growing plastic industry until 2016, when plastic bags with the thickness below 50 μm were banned [82].

It is recommended that policies focus on secondary microplastics, such as microfibers, because the major source of microfibers is the washing of clothes [45]. Policies should be developed to improve domestic wastewater treatment processes to filter out microfibers, as waste management policies directly influence microplastic abundance in the environment [83]. Furthermore, policies for regular monitoring of microplastic abundance in various ecosystems have been suggested [82]. In addition to reducing plastics from consumption or import, and capturing microplastics before they contaminate the natural environment, more studies are required to further discuss the heterogeneity of human activities and how to effectively control microplastic pollution. Recently, a few studies have attempted to further discuss different kinds of human activities based on land-use-survey data. They have delineated watershed margins, presuming that microplastics released from sources are mainly transported by rain runoff to rivers or lakes (freshwater systems), and incorporated a land-cover dataset to represent distinctive human activities. These studies have shown that industrial/urban areas within catchments are potential sources of microplastics. However, future challenges in this context will be to answer the following questions: (1) Can the resolution of land-cover data increase so that we can identify the exact industry producing the largest amount of microplastics in industrial areas, and can the resolution of microplastic properties increase (with better microplastic identification efficiency) so that we can identify a specific “marker” substance of microplastics that may be representative of a certain type of industry? (2) Because watershed margin delineation has a huge influence on the results, is the watershed margin reliable if river channels are artificially manipulated in urban river systems, and should the sewer system be considered? (3) Are there better approaches to quantifying anthropogenic activities that can help the government build related policies?

In conclusion, these challenges can be tackled by the construction of accessible and reliable land-cover surveillance data from government agencies, the development of better microplastic-substance-identification techniques and protocols, and the available data on artificial river channels and sewer systems in urban areas. Although the relationship between anthropogenic activities and microplastics is yet to be comprehensively studied, this review has observed positive and consistent progress on this issue, showing that these problems are expected to be addressed in the near future. This review integrates the global literature on environmental microplastics and anthropogenic activities, highlighting the necessity of summarizing results with statistical analyses. It is imperative to quantify anthropogenic factors by using indices other than population density, which creates equivocal results whose use by policymakers is difficult. At present, this review suggests that using watershed-scale attributes derived from land-use datasets might produce a more in-depth scientific basis for government authorities and environmental protection organizations and institutions to articulate efficient policies to reduce microplastic pollution.
